# Gait parameters as tools for analyzing phenotypic alterations of a mouse model of Charcot-Marie-Tooth disease

**DOI:** 10.1080/19768354.2021.1880967

**Published:** 2021-02-11

**Authors:** Sun Hee Hwang, Eun Hyuk Chang, Geon Kwak, Hyeonjin Jeon, Byung-Ok Choi, Young Bin Hong

**Affiliations:** a Department of Neurology, Samsung Medical Center, Sungkyunkwan University School of Medicine, Seoul, Republic of Korea; b Samsung Biomedical Research Institute, Samsung Advanced Institute of Technology, Samsung Electronics Co., Ltd., Seoul, Republic of Korea; c Department of Health Sciences and Technology, SAIHST, Sungkyunkwan University, Seoul, Republic of Korea; d Department of Biochemistry, College of Medicine, Dong-A University, Busan, Republic of Korea; e Department of Translational Biomedical Sciences, Graduate School of Dong-A University, Busan, Korea

**Keywords:** Biomarker, Charcot-Marie-Tooth disease, gait analysis, mouse model

## Abstract

Charcot-Marie-Tooth disease (CMT), a genetically heterogeneous group of diseases in the peripheral nervous system, is characterized by progressive and symmetrical distal weakness resulting in gait abnormality. The necessity of the diagnostic and prognostic biomarkers has been raised for both basic research and clinical practice in CMT. Since biomarkers for animal study of CMT are limited, we evaluated the feasibility of gait parameters as tool for measuring disease phenotype of CMT mouse model. Using a Trembler-J (Tr-J) mouse, a CMT type 1 (CMT1) mouse model, we analyzed kinematic parameters such as angles of hip, knee and ankle (sagittal plane), and spatial parameters including step width and stride length (transverse plane). Regarding of kinematic parameters, Tr-J mice exhibited less plantarflexed ankle during the swing phase and more dorsiflexed ankle at the terminal stance compared to control mice. The range of motion in ankle angle of Tr-J mice was significantly greater than that of control mice. In spatial parameter, Tr-J mice exhibited wider step width compared to control mice. These results are similar to previously reported gait patterns of CMT1 patients. In comparison with other markers such as nerve conduction study and rotarod test, gait parameters dynamically reflected the disease progression of CMT1 mice. Therefore, these data imply that gait parameters can be used as useful tools to analyzed the disease phenotype and progression during preclinical study of peripheral neuropathy such as CMT.

## Introduction

Charcot-Marie-Tooth disease (CMT) is a genetically heterogeneous group of diseases in the peripheral nervous system characterized by progressive and symmetrical distal weakness (Harding and Thomas 1980). The symptoms of CMT appear in a length dependent manner (Vinci 2003); as the peripheral nerves degenerate, the distal muscles gradually become atrophied and weaken from the distal to the proximal limbs. Thus the characteristic features appear in gait disturbance.

Assessments of the gait abnormality caused by muscle loss and foot deformity indicated that CMT patients exhibited the push-off deficit, foot-drop, increased foot supination, excessive internal rotation of the knee and/or tibia, knee hyperextension during the stance phase, excessive external rotation at the hip, and decreased hip adduction in stance (Don et al. 2007; Newman et al. 2007). According to Ferrarin et al. the gait pattern of children patients with CMT type 1A (CMT1A) can be classified into three clusters: pseudo-normal patients, patients showing only footdrop, and patients with footdrop and push-off deficits (Ferrarin et al. 2012). In a follow-up study, they reported that the footdrop and push-off deficit presented as significant changes over 18 months in the CMT1A patients (Ferrarin et al. 2013). Moreover, a fatigue and myometry study showed hip flexor fatigue caused by compensation of distal weakness in CMT patients (Ramdharry et al. 2009).

To date, clinical parameters for peripheral neuropathy have been similarly applied to preclinical researches. Electrophysiological examination, muscle integrity measured by grip strength and rotarod test, and several sensory tests are the examples. However, several parameters have shown the bottom effect which deteriorates the sensitivity of the parameters in both CMT patients and animal models. Thus the development of new biomarkers is required for both preclinical study and clinical practice.

Gait analysis can be a candidate option in that it provides various kinematic and spatiotemporal parameters which are correlated with muscle integrity or motor function coordination. In addition, the analysis can be executed in natural environment without any invasive procedure. Most frequently applied analysis in animal gait is foot printing which can indicate the muscle function based on step width and clarity of the footstep. However, this analysis has limitation in the kinematic parameters which indicate the integrity of each muscle based on the joint angles. In this study, we assessed the feasibility and applicability of the kinematic gait parameters for CMT mouse model using a newly developed apparatus.

## Materials and methods

### Mouse and phenotype evaluation

Trembler-J (Tr-J) mouse (B6.D2-Pmp22Tr-J/J, Jackson laboratory, Bar Harbor, ME) was used as a CMT type 1 (CMT1) mouse model (Henry et al. 1983). All experiments were conducted according to protocols approved by the Institutional Animal Care and Use Committees of Samsung Medical Center. Evaluation of CMT1 phenotype was performed according to previous study (Lee et al. 2015). Assessment of motor coordination and balance were performed using an apparatus (B.S. Technolab INC, Seoul, Korea) with a horizontal rotating rod (21rpm). The maximum holding time of mice on the rotating rod was recorded up to 300 s. Nerve conduction study (NCS) was performed to determine motor nerve conduction velocity (MNCV) and compound muscle action potential (CMAP) using a Nicolet VikingQuest device (Natus Medical, San Carlos, CA) after the mice were anesthetized with 1.5% isoflurane supplied using a nose cone (Lee et al. 2019).

### Gait analysis

For determination of gait pattern of mouse model, we applied MotoRater system (TSE systems, Bad Homburg, Germany). The system has a narrow channel made of clear acryl for mouse walking with two mirrors on both side of the channel to reflect the motion of lateral side of the mouse, and a mobile high-speed camera, which automatically follows and records the mouse movements from three sides simultaneously. Gait patterns such as the angles of hip, knee and ankle (sagittal plane) as well as step width and stride length (transverse plane) were used. To indicate their locations, the hair was removed and each joint was marked. Iliac crest, center of hip, knee and ankle joints, 5th metatarsal head of right side, which are regarded as center of the joints, were marked using white marking pen to calculated hip, knee and ankle joints. From the recorded images, the marked points on the right side were tracked in each frame to generate the coordination of each joint during walking.

### Parameters and data analysis

**Figure 1.**
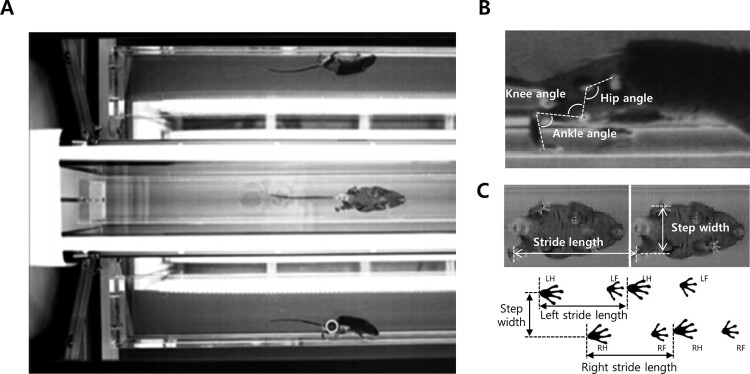
MotoRator system and description of parameters for mouse gait analysis. (A) MotoRator system has a narrow channel with two mirros on both side of the channel to monitor lateral side of the mouse. (B) Description of sagittal plane parameters including angles of each hip, knee, and ankle. (C) Description of transverse plane parameters step width and stride length.

After recording the gait of each mouse, we then analyze the gait pattern using each parameter. First, we extracted the middle two steps from the whole recorded gait. This enables the analysis of steady-state gait pattern by exclusion of accelerating and decelerating periods during the whole gait. To compare kinematic patterns between Tr-J and wild-type (WT) mice, the x and y coordinates for 5 marked points were extracted on the sagittal plane video using MotoRater software (Figure 1). Then the positions of each 5 point were analyzed with MATLAB 2010a (MathWorks Inc., USA). The law of cosines was used to calculate the angles using the Euclidean distances of three points. Using this, the angles of each hip, knee and ankle were determined by measuring the distances between three marked points. Then the gait pattern of each mouse were analyzed by comparing the changes of each kinematic parameter of the hip, knee, and ankle angles during a gait cycle. A gait cycle was defined as a period from the first ground strike of right hindpaw to the next touch of same limb to the ground, which corresponds to the human gait analysis. Then the range of motion (ROM) in three joints (hip, knees, and ankle), which indicate the difference between minimum and maximum values during a gait cycle, were analyzed after determination of minimum and maximum joint angles. The temporal–spatial parameters such as stride length and step width were analyzed in the transverse plane. The stride length was determined by measuring the distance of the hindpaw shift from previous and next step during a gait cycle. The step width was calculated by measuring the distance between two hindpaws during stance phase (Figure 1).

### Statistical analyses

All values are expressed as mean ± standard error of the mean (SEM). The statistical significance of data presented were evaluated by Student’s *t*-test. The level of significance was set to *p *< 0.05.

## Results

### Joint angle changes during the disease progression in CMT1 mice

In this study, we used Tr-J mouse which harbors a naturally occurred point mutation (p.Leu16Pro) in *PMP22* gene. Tr-J mouse develops CMT1 phenotype afterbirth and definite phenotypic abnormality appeared within 3 weeks of age. For determination of gait pattern of mouse model, we applied a MotoRater system which allows the record of the mouse motion from 3 sides simultaneously (Figure 1(A)). To acquire several gait parameters including the angles of hip, knee and ankle (sagittal plane) as well as step width and stride length (transverse plane) (Figure 1(B,C)), we recorded the mouse gait and calculated for each parameter by measuring the movements of each marked position.

**Figure 2.**
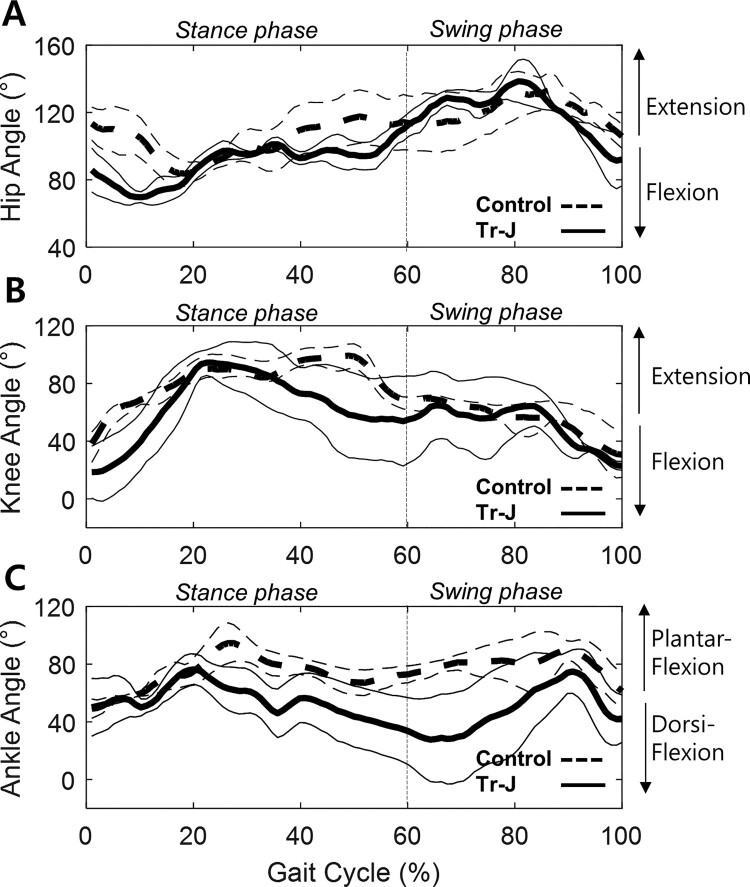
Kinematic data of Tr-J (*n* = 5) and WT mice (*n* = 5) at 14 weeks of age. (A) Hip angle (B) Knee angle (C) Ankle angle. Solid line, Tr-J mice; dashed line, WT mice. Thin lines, ± 1 standard deviation of Tr-J (solid) and WT (dashed) mice.

Gait analysis for five (three females and two males) of each group (Tr-J and WT) mice were performed at 6, 10 and 14 weeks of ages. First, we compared the kinematic patterns in sagittal plane at 14 weeks of age. Hip angles of Tr-J mice were more fluctuated compared to those of WT mice (Figure 2(A)). Maximum and minimal hip angle, which correspond to maximum hip extension and flexion angles, respectively, were greater than control. As the result, the ROM of hip joint was greater than WT mice, which had also been observed in CMT1A patients (Don et al. 2007). According to knee angle comparison, Tr-J mice showed more flexed than controls (Figure 2(B)). Since muscle weakness in CMT1 begins from the distal limbs, gait disturbances are initially perceived in the ankle angle. The minimum and maximum ankle angle of Tr-J mice were significantly different from WT mice thereby the range of motion (ROM) in Tr-J was also greater than WT mice (Figure 2(C)). Tr-J mice had less plantarflexed ankle during the swing phase than controls. It showed more dorsiflexed ankle at the terminal stance, around 60% of gait cycle compared to WT mice, although Tr-J mice are supposed to have dorsiflexor weakness. These results might be attributed to the passive dorsiflexion due to the weakness of plantarflexor, an antagonist for dorxiflexor: In the terminal stance, the plantarflexors need to be activated to push off, yet the weakened plantarflexors failed to push off in Tr-J mice.

**Figure 3.**
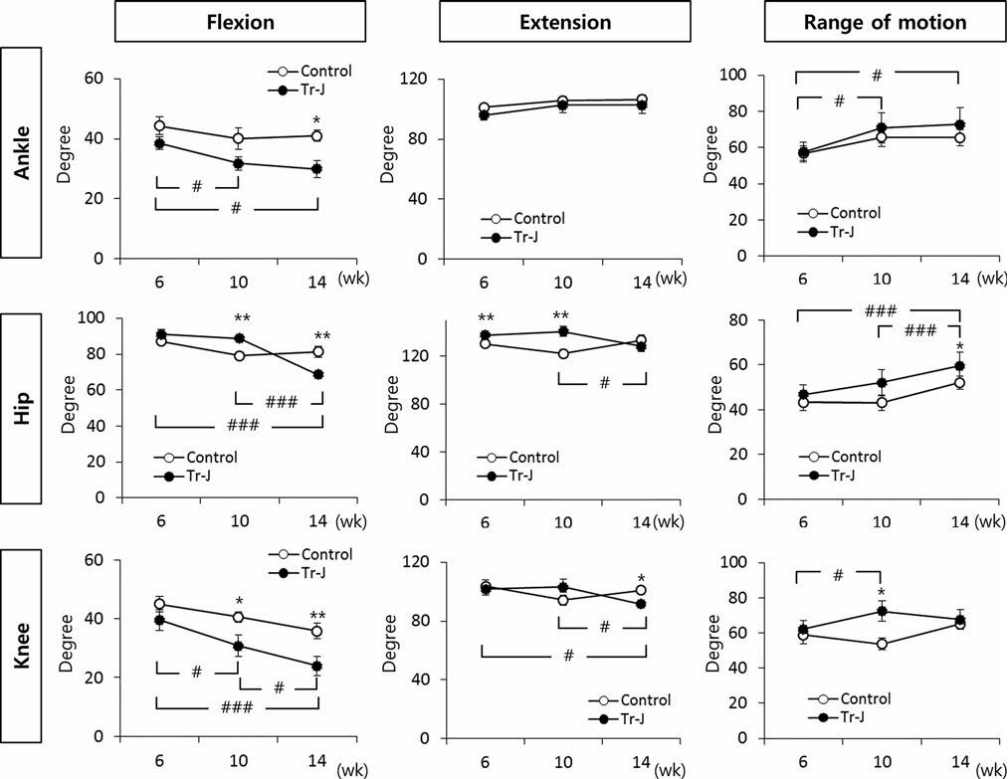
Follow-up data of sagittal plane parameters. * represent statistical significance between Tr-J and WT mice in the same time point: *, *p < *0.05; and **, *p < *0.01. # represents statistical significance between time points within same mouse group: ^#^, *p < *0.05 and ^###^, *p < *0.001

Next, we sought to correlate the disease progression with gait abnormality. When the kinematic parameters were compared in time course from 6 to 14 weeks, flexion, extension and ROM of each angle were significantly altered according to disease progression of Tr-J mice (Figure 3). Dorsiflexion angle of ankle appeared greater in Tr-J mice than WT mice and more dorsiflexed in a time dependent manner. The hip angle also showed significantly more flexed and extended up to 10 weeks resulting in greater ROM in Tr-J mice compared to WT mice. On the other hand, Tr-J mice showed less flexed in knee joint as the disease progressed.

The primary signs of CMT1A patients are foot drop and/or push-off deficit, which are related to ankle angle. In follow-up evaluation, patients showed worsened foot-drop and exaggerated hip flexion than previous test (Ferrarin et al. 2013). Similarly, ankle angle of Tr-J mice from the early state of disease was significantly different compared to WT. The knee and hip angles between two mice groups even more changed in a time dependent manner in order to compensate distal muscle weakness and the disease progression to proximal muscles. Therefore, the flexion angles of knee and hip in Tr-J mice were evidently weaker in 14 weeks.

### Longer stride length and wider step width in CMT1 mice

**Figure 4.**
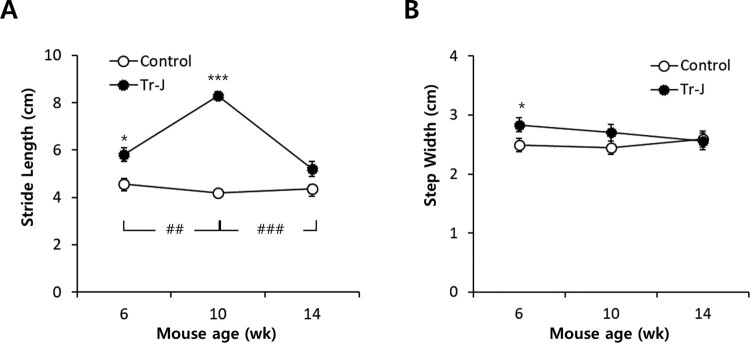
Follow-up of transverse plane parameters. (A) Stride length. (B) Step width. * represents the statistical significance between Tr-J and WT mice in the same time point were analyzed: *, *p < *0.05 and ***, *p* <0.001. ^#^ represents statistical significance between time points within same mouse group: ^##^, *p < *0.01 and ^###^, *p < *0.001

Since several kinematic data between Tr-J and WT mice were significantly different, we hypothesized that the temporal–spatial parameters in sagittal plane might also be altered in Tr-J mice. Then we analyzed the transverse plane to determine step width and stride length. The stride length of Tr-J mice was significantly longer than that of the control mice (Figure 4(A)). Specifically, the most difference appeared at 10 weeks and it was recovered at 14 weeks. Previously, the CMT1A patients show shorter stride length than controls, which caused by muscle weakness and balance disturbances (Don et al. 2007). The longer stride length in Tr-J mice might be derived from the selective weakness of dorsiflexor muscles (Chung et al. 2008). The accompanied weakness of dorsiflexor by plantarflexor results in the extension of stance phase, which corresponds to the difficulty of push-off and foot clearance in CMT patients. To compensate the distal muscle weakness and the leak of foot clearance, the higher ROMs in hip and knee angles presented in CMT patients. As the disease becomes severe, the proximal muscles eventually become weaker, and the ambulatory function of the mice cannot be compensated.

The step width in Tr-J mice was wider than WT mice (Figure 4(A)), which was also observed in human CMT patients (Don et al. 2007). The wider step width, which indicates a broader support area, is considered to be related to balance disturbances. According to the patients’ data, the characteristics of gait in CMT patients were subdivided as two groups: steppage gait and clumsy pattern. The former is characterized by reduced swing velocity and preserved step length in spite of a high energy consumption. On the other hand, the latter showed very slow gait with reduced step length, a broader support area and great reduction in the cadence. The difference between two group might be induced by both primary motor deficits and secondary compensation mechanisms (Don et al. 2007).

### Sensitivity of gait parameter for disease diagnosis

**Figure 5.**
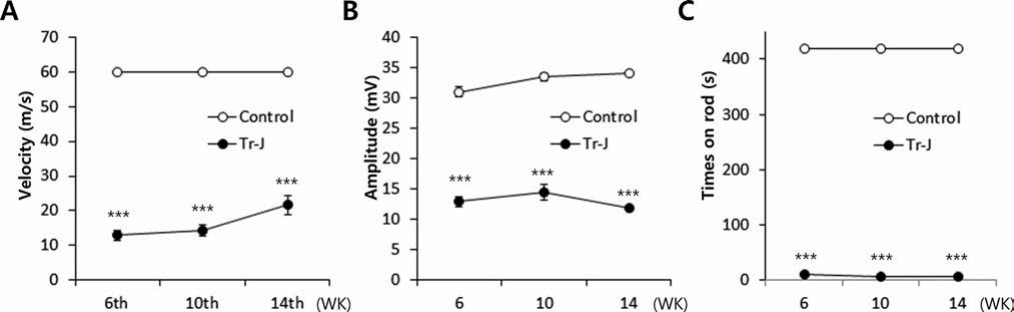
Nerve electrophysiologic parameters and rotarod performance data of Tr-J (*n* = 5) and WT mice (*n* = 5). (A) Motor nerve conduction velocity (MNCV). (B) Compound muscle action potential (CMAP) (C) Rotarod performance. *, *p <* 0.001.

To evaluate whether gait parameters are sensitive to diagnose disease progression in CMT1 mouse model, we compared the gait data with electrophysiology and locomotor performance data. First, we compared the nerve electrophysiology on sciatic nerve. As Tr-J mouse exhibits the phenotypic defects within a month from birth, the peripheral nerve defects arise from developing stages. Both motor nerve conduction velocity (MNCV) and compound muscle action potential (CMAP) of Tr-J mice were significantly lower than WT mice from the first observation (6 weeks of age). In addition, there were very slight changes according to the disease progression in Tr-J mice (Figure 5(A,B)). Next, we compared the rotarod performance in both mice. In accordance with the definite phenotypic difference, the performance was significantly lower than WT mice in all the observation time points (Figure 5(C)). These data imply that the parameters from electrophysiology and locomotor performance showed the bottom effect, thereby losing the sensitivity to CMT1 mouse model.

## Discussion

In this study, we seek the answers whether gait analysis would become a new parameter to diagnosis or to measure the disease progression of hereditary peripheral neuropathy in mouse models. Using a gait monitoring system, we analyzed gait parameters such as angles of hip, knee and ankle (sagittal plane), step width and stride length (transverse plane). Gait disturbances of CMT1 mice were significantly different to that of WT mice in their early stage of disease. Distal muscle weakness induced changes in ankle angle, which affects to the patterns of knee and hip joint angle for the compensation. These data mostly coincide with CMT1A patients pattern except that CMT1A patients show dorsiflexor weakness in the early stage, then it carried over into the plantarflexor weakness (Table 1). In addition, CMT1 mice exhibited longer stride length, which can be explained by dorsiflexor weakness. The delayed toe-off due to dorsiflexor weakness may result in longer step length. Although the causes are the same in human and mouse, the results can differ due to biped gait patterns and the quadruped foot gait. On the other hand, CMT1 mice showed the wider step width compared to WT mice, which is in accordance with CMT1 patients (Don et al. 2007).

**Table 1.** Summary of gait parameters in Tr-J mice and comparison with CMT1A patients

Parameters	Tr-J mice (compared to WT mice)	CMT1A patients (compared to normal)
**Ankle angle**		
Dorsiflexion	more flexed	more flexed
Plantarflexion	less flexed	less flexed
ROM	N.D.	smaller
**Hip angle**		
Flexion	more flexed → less flexed	more flexed
Extension	N.D.	N.D.
ROM	more flexed	N.D.
**Knee angle**		
Flexion	more flexed	N.D.
Extension	N.D.	N.D.
ROM	N.D.	N.D.
Step width	wider	wider
Stride length	longer	shorter

Compared to the parameters from nerve conduction or locomotor performance, gait analysis exhibited more effective features to monitor disease progression. Long-term follow-of gait analysis with identical mice demonstrated the usefulness of gait analysis as a sensitive biomarker in disease diagnosis. Although electrophysiological analysis and rotarod performance showed a significant difference between Tr-J and WT mice, the parameters were undistinguishable within time sequences due to bottom effect. Indeed, the electrophysiological data were almost at the borderline of measuring range and the times on rod were close to zero from the first examination in Tr-J mice.

In the clinical research for efficacy assessment of novel therapeutics, development of useful biomarker is prerequisite. However, very slow rate of disease progression causes very slow changes in available parameters. According to previous studies, the average disease progression determined by CMT neuropathy score (CMTNS) is rather limited (Shy et al. 2008; Verhamme et al. 2009b), and the biomarkers used in the clinical trials also exhibited limited values (Micallef et al. 2009; Verhamme et al. 2009a; Lewis et al. 2013; Attarian et al. 2014). Our results also indicated the very slow progression in MNCV and CMTNS. Thus these small ranges of changes have been a concern for natural history studies and clinical trials in CMT.

In preclinical study, parameters for drug efficacy need to reflect the human phenotypic symptoms. So far, no satisfactory outcome has been drawn from clinical studies, even though several treatment options for CMT have been suggested through animal studies. For example, application of ascorbic acid showed dramatic efficacy in rodent models (Passage et al. 2004; Kaya et al. 2007), however, the efficacy was proved to be strictly limited in a couple of clinical trials (Pareyson et al. 2011; Lewis et al. 2013). These data imply several limitations in the bridging the preclinical study to clinical outcome: First, the biomarkers used in the preclinical study may not be suitable to clinical study. Second, there might be phenotypic difference between the CMT patients and the rodent models. Therefore, the development of preclinical biomarkers akin to those from the patients is required. In these contexts, gait analysis might be a potent candidate to monitor the minute disease progression.
